# Variation of the modulus of elasticity of aligner foil sheet materials due to thermoforming

**DOI:** 10.1007/s00056-021-00327-w

**Published:** 2021-08-19

**Authors:** Bijan Golkhani, Anna Weber, Ludger Keilig, Susanne Reimann, Christoph Bourauel

**Affiliations:** 1 Private Practice, Frechen, Germany; 2 Oral Technology, University of Bonn, Bonn, Germany; 3 Oral Technology and Department of Prosthodontics, Preclinical Education and Dental Materials Science, University of Bonn, Bonn, Germany; 4 University of Applied Sciences, Bremerhaven, Germany; 5 Oral Technology, University of Bonn, Welschnonnenstr. 17, 53111 Bonn, Germany

**Keywords:** Orthodontic appliances removable, Thermoformed splints, Mechanical properties, Three-point bending, Young’s modulus, Herausnehmbare kieferorthopädische Geräte, Tiefgezogene Schienen, Mechanische Eigenschaften, Drei-Punkt-Biegung, Elastizitätsmodul

## Abstract

**Objective:**

Investigate and compare the mechanical properties of different aligner materials before and after deep drawing and determine differences in the mechanical properties after thermoforming.

**Materials and methods:**

Four aligner film sheets from three manufacturers (Duran Plus® [Scheu Dental, Iserlohn, Germany]; Zendura® [ClearCorrect, Bay Materials LLC, Fremont, CA, USA]; Essix ACE® and Essix® PLUS™ [Dentsply Sirona Deutschland, Bensheim, Germany]) were tested in 3‑point bending with support distances of 8, 16, and 24 mm. Dimension of the specimens was 10 × 50 mm^2^. Two groups each were tested: (1) 10 specimens were investigated in the as-received state (before thermoforming), (2) 10 specimens were deep drawn on a master plate with cuboids of the dimension 10 × 10 × 50 mm^3^. Then, specimens were cut out of the upper side and lateral walls and were measured in 3‑point bending. Forces and reduction in thickness were measured and corrected theoretical forces of drawn sheets after thickness reduction as well as Young’s modulus were calculated.

**Results:**

At a support distance of 8 mm and a displacement of 0.25 mm Essix® PLUS™, having the highest thickness in untreated state, showed highest forces of 28.2 N, followed by Duran Plus® (27.3 N), Essix ACE® (21.0 N) and Zendura® (19.7 N). Similar results were registered for the other distances (16, 24 mm). Thermoforming drastically reduced thickness and forces in the bending tests. Forces decreased to around 10% or less for specimens cut from the lateral walls. Young’s modulus decreased significantly for deep drawn foil sheets, especially for Essix® PLUS™.

**Conclusions:**

Three-point bending is an appropriate method to compare different foil sheet materials. Young’s modulus is significantly affected by thermoforming.

**Supplementary Information:**

The online version of this article (10.1007/s00056-021-00327-w) contains supplementary information, which is available to authorized users.

## Abstract (de)

**Ziel:**

Untersuchung und Vergleich der mechanischen Eigenschaften verschiedener Aligner-Materialien vor und nach dem Tiefziehen und Ermittlung von Unterschieden in den mechanischen Eigenschaften nach dem Warmverformen.

**Materialien und Methoden:**

Vier Alignerfolien von 3 Herstellern (Duran Plus® [Scheu Dental, Iserlohn, Deutschland]; Zendura® [ClearCorrect, Bay Materials LLC, Fremont/CA, USA]; Essix ACE® and Essix® PLUS™ [Dentsply Sirona Deutschland, Bensheim, Deutschland]) wurden im 3‑Punkt-Biegeversuch mit Stützpunktabständen von 8, 16 und 24 mm vermessen. Die Abmessungen der Probekörper betrugen 10 × 50 mm^2^. Es wurden jeweils 2 Gruppen getestet: 1) 10 Proben wurden im Ausgangszustand (vor dem Tiefziehen) untersucht. 2) 10 Proben wurden über eine Schablone mit Quadern der Abmessung 10 × 10 × 50 mm^3^ tiefgezogen. Dann wurden aus der Oberseite und den Seitenwänden Proben geschnitten und in 3‑Punkt-Biegung vermessen. Kräfte und Dickenreduktion wurden gemessen und korrigierte theoretische Kräfte der gezogenen Folien nach Dickenreduktion sowie der Elastizitätsmodul berechnet.

**Ergebnisse:**

Bei einem Stützpunktabstand von 8 mm und einer Auslenkung von 0,25 mm zeigte Essix® PLUS™ mit der größten Dicke im unbehandelten Zustand die höchsten Kräfte von 28,2 N, gefolgt von Duran Plus® (27,3 N), Essix ACE® (21,0 N) und Zendura® (19,7 N). Vergleichbare Ergebnisse wurden für die anderen Abstände (16, 24 mm) registriert. Durch Thermoformen wurden die Dicke und die Kräfte in den Biegeversuchen drastisch reduziert. Bei Proben, die aus den Seitenwänden geschnitten wurden, verringerten sich die Kräfte auf etwa 10 % oder noch weniger. Der Elastizitätsmodul nahm bei tiefgezogenen Folien deutlich ab, insbesondere bei Essix® PLUS™.

**Schlussfolgerungen:**

Der 3‑Punkt-Biegeversuch ist eine geeignete Methode, um verschiedene Folienmaterialien zu vergleichen. Der Elastizitätsmodul wird durch das Thermoformen erheblich beeinflusst.

## Introduction

In recent years, orthodontic therapy using removable, clear aligners has become very popular and increasing numbers of patients request this almost invisible treatment option. The first thermoplastic appliance was described in 1945 by Kesling [11]. Tooth movement was achieved without bands, brackets or wires. Later others reported about different types of thermoformed splints, such as invisible retainers [18]. Typical aligner materials are polyethylene (PE), polyethylene terephthalate (PET), polyethylene terephthalate glycol (PETG), polyurethane (PU) and polypropylene (PP) with further modifications of vinyl acetate and polyurethane aiming to increase the patient’s comfort [6, 28]. In recent years, removable thermoplastic appliances were used for several treatments, e.g., retainers, night guards, bleaching, temporomandibular joint (TMJ) splints [9, 16, 19, 22, 23].

For almost 20 years now, removable thermoplastic appliances proved to be a good alternative to conventional bracket and arch wire appliances if certain restrictions are considered. According to a 2010 statement of the German Society of Orthodontics [5] aligners are suitable means to correct moderate anterior crowding or interdental spacing, anterior protrusion and retrusion as well as minor in- or extrusion by using attachments. Further tooth movements are possible if additional auxiliaries are applied. According to the statement, contraindications cannot be defined if aligners are used in combination with other techniques to solve subtasks. Thermoplastic appliances have several beneficial aspects compared to conventional orthodontic appliances, such as better oral hygiene, less plaque accumulation, and easy usage by the patient. Aligner therapy is used mainly for correction of malposition of canines and incisors [17, 20, 22, 27]. The working principle of such appliances is based on the deviation between the actual tooth position and a setup position which is produced as the negative shape of the aligner. The programmed geometry of the splint then defines the new tooth position and the amount of movement to be performed [16].

Two basically different concepts can be identified in aligner treatment with respect to the tooth movement per splint. The first concept uses a larger number of subsequent splints with small setup increments between 0.1 and 0.2 mm (e.g., Align Technology, Inc., San Jose, CA, USA). The alternative concept is using larger setup increments of 0.5–1.0 mm and materials with increased elasticity, such as the Clear Aligner System (Scheu Dental GmbH, Iserlohn, Germany) [2, 12]. It is important to minimize forces by proper selection of aligner material and aligner stepping, as apical root resorption may result from heavy forces even in aligner orthodontics [13].

Additionally aligners can be used for finishing of orthodontic treatment with small, final corrections [5]. However, today, according to manufacturers and several studies, aligners can effectively perform major tooth movements, such as bicuspid derotation of up to 50° or root movement of upper central incisors of up to 4 mm [1, 25].

The raw material for individualized aligner production is offered by several companies. However, little is known about material parameters and change of material parameters after thermoforming. In addition, no standardized test according to an ISO or national standard has been proposed until now for the measurement of material characteristics of aligner sheets. Only a few papers reported on comparative aligner sheet measurements [14, 21]. Several studies reported that mechanical and physical properties of orthodontic aligner materials may change after thermoforming, cyclic mechanical loading and/or thermocycling processes as well as after clinical use [3]. Thus mechanical properties should be determined after thermoforming and under the influence of thermocycling and cyclic loading [2, 8, 10, 21].

The purpose of this research was to investigate the mechanical properties of different aligner raw materials, delivered as thermoforming sheets with varying thickness before and after deep drawing. The influence of test geometry, the amount of thickness reduction and the change of material parameters due to drawing over a three-dimensional (3D) object were to be analyzed as well. Test geometries described by Elkholy et al. [8] and Kwon et al. [14] were used.

## Materials and methods

### Tested materials and preparation

**Fig. 1:**
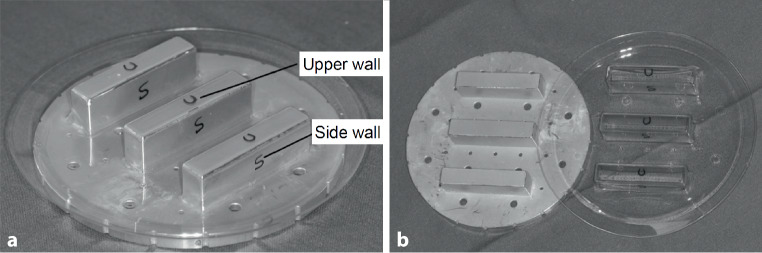
**a** Master plate for deep drawing of the specimens from the foil sheets. **b** Specimens cut from the upper side and the side wall were marked with an *U* and an *S*, respectively **a** Tiefziehschablone zur Herstellung der Proben aus den Alignerfolien. **b** Aus der Oberseite und der Seitenwand geschnittene Proben wurden mit einem *U* bzw. einem *S* markiert

Four sheet films for the production of aligners from three manufacturers were tested. All information regarding manufacturer, product name, material composition, and sheet film dimensions are listed in Table 1. All materials were of the single-layer type and were selected with a diameter of 125 mm and a thickness of 0.75 mm, except Essix® PLUS, with 0.90 mm thickness. Two different test series were performed:

- I—Untreated specimens (raw material): 10 specimens (dimension: 50 × 10 mm^2^) for each material were prepared for 3‑point bending tests. The sheets remained in its as-received state.
- II—Deep drawn specimens: A second test group with identical dimensions was prepared by deep drawing of the sheets according to manufacturer’s instructions. A master plate made of aluminum (Fig. 1a) was used, consisting of a perforated plate and 3 cuboids in the center. Thermoforming was performed in a commercial device (Ministar S, Scheu, Iserlohn, Germany). All thermoforming processes were performed strictly according to manufacturers’ recommendations regarding pressure, heating, and cooling time. Details of the manufacturers’ instructions are listed in Table 1. A cooling period for master plate of 20–25 min was strictly kept between each thermoforming process in order to ensure no uncontrolled thermoforming prior to insertion of the foil sheets into the Ministar device. As recommended by the manufacturer, the maintainer foil of Duran Plus® was removed after thermoforming.

**Table 1:** Tested products with manufacturers’ names, composition, physical dimensions and thermoforming parameters Untersuchte Produkte mit Herstellernamen, Zusammensetzung, physikalischen Abmessungen und Tiefziehparametern

Product Name	Manufacturer	Composition	Sheet film diameter, mm	Thickness, mm	Heating time, s	Cooling time, s	Pressure, bar
Duran Plus®	Scheu Dental GmbH, Iserlohn, Germany	PET‑G	125	0.75	20	30	4
Zendura®	ClearCorrect, Bay Materials LLC, Fremont, CA, USA	Polyurethane	125	0.75	40	60	4
Essix ACE®	Dentsply Sirona Deutschland GmbH, Bensheim, Germany	Copolyester	125	0.75	25	30	4
Essix® PLUS™	Dentsply Sirona Deutschland GmbH, Bensheim, Germany	Copolyester	125	0.90	35	50	4

After removing the sheet from the plate, 10 specimens each were cut out of the “upper side” (marked ‘U’) and the side of the cuboids (‘S’). Dimensions of the specimens were identical to test I (50 × 10 mm^2^, Fig. 1b). Cutting of all specimens was done at room temperature using scalpel and scissors. Care was taken to remove all cutting burrs using sandpaper (grain size 240 and 500).

Thickness of the drawn sheets was determined at three positions (left, central, right in the middle part of specimens) with a digital caliper (Alpha Tools, Bahag, Mannheim, Germany) before and after thermoforming to document thickness changes.

## Test geometry

**Fig. 2:**
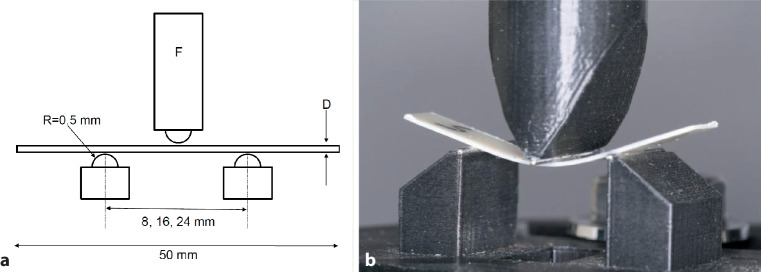
**a** Test geometry of the 3‑point bending test. The distance of the support points could be varied between 8, 16 and 24 mm. **b** Round steel wires with a diameter of 1 mm were used for the supports and the thrust die. *F* force applied through the thrust die, *D* thickness of the specimen, *R* radius of the support points **a** Geometrie des 3‑Punkt-Biegeversuchs. Der Abstand der Stützpunkte konnte zwischen 8, 16 und 24 mm variiert werden. **b** Für die Stützpunkte und die Druckfinne wurden Rundstahldrähte mit einem Durchmesser von 1 mm verwendet. *F* durch die Druckfinne aufgebrachte Kraft, *D* Dicke der Probe, *R* Radius der Stützpunkte

Mechanical properties of the foil sheets were investigated using a standardized 3‑point bending test setup (Fig. 2), integrated into a universal materials testing machine (Zwick/Roell ZmartPro, Zwick, Ulm, Germany). Radius of curvature of the supports and the thrust die were 1 mm. Distances of the support points were 8, 16, and 24 mm, as described by Kwon et al. [14] and Elkholy et al. [8]. Central displacements at these distances were 0.25, 0.50, and 2.00 mm, respectively, and were taken from the same studies [8, 14]. Displacements were selected such that no plastic deformation or microcracks were traceable by visual inspection of the material.

### Theoretical forces of as-received and drawn sheets with compensated thickness reduction

Theoretical forces of foil sheets were calculated using the formula of 3‑point bending:

1 $$\mathrm{F}=48\cdot \mathrm{E}\cdot \mathrm{I}\cdot \frac{\mathrm{d}}{\mathrm{L}^{3}}$$

where F = generated force, E = Young’s modulus of the material, d = central displacement, L = distance between the support points and I = moment of inertia:

2 $$\mathrm{I}=\frac{\mathrm{b}\cdot \mathrm{h}^{3}}{12}$$

where b = width of the specimens and h = measured thickness. For the as-received sheets the following approach was used: Values for Young’s modulus stated in literature and in manufacturers’ data sheets were reviewed and minimum as well as maximum values were recorded for E measured in tension and bending [21, 26]. The height h (in Eq. (2)) was taken from the actual measurements of the as-received foil sheets. The range of the theoretical forces of the four foil sheet types were calculated using Eqs. (1) and (2) together with the d and L values from the three set-up configurations and the Young’s modulus determined above.

In addition, a compensation of thickness reduction due to thermoforming was done by calculating the cube of the proportion of the as-received thickness and the deep drawn thickness. Then the corrected force was calculated from the measured force of the deep drawn specimens by multiplying it with this proportion. Finally, experimental Young’s modulus was determined for all sheets in as-received and thermoformed state by solving the Eq. 1 for E.

### Data processing

Mean and variance of all values were calculated from the raw data. Graphical presentation was done as box/whisker plots. As only a minor part of the data was not normally distributed (Shapiro–Wilk test), Student’s t‑tests with Bonferroni correction for multiple testing were performed to test for significance with a P level set to 5%. Data processing, statistical analysis and presentation were performed using PlotIT for Windows (Scientific Programming Enterprises, Haslett, MI, USA) and Excel (Microsoft Corporation, Redmond, WA, USA).

## Results

### Thickness reduction after thermoforming

**Fig. 3:**
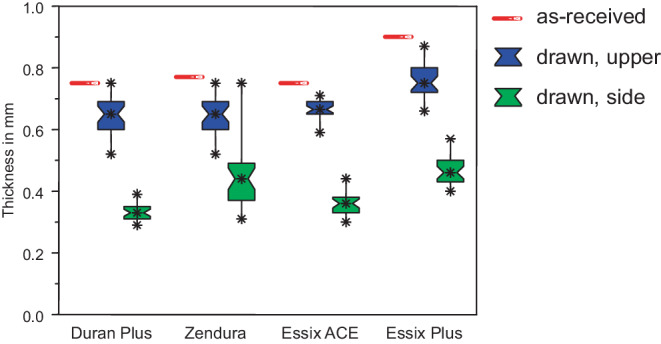
Thickness of the tested materials before and after deep drawing. Reduction in thickness was significantly higher for the side walls compared to specimens from the upper side. *Red* as-received, *blue* upper side, *green* side walls Dicke der getesteten Materialien vor und nach dem Tiefziehen. Bei den Seitenwänden war die Dickenreduktion signifikant höher als bei den Proben aus der Oberseite. *Rot* unbehandelt, *blau* Oberseite, *grün* Seitenwände

Due to the drawing process, thickness of the sheets was clearly reduced. Measured thicknesses of the as-received and deep drawn sheets are summarized in Fig. 3. The thickness of the ‘U’-type specimens were as follows (mean and standard deviation): Duran Plus® 0.65 (0.06) mm, Zendura® 0.65 (0.06) mm, Essix ACE® 0.66 (0.03) mm, and Essix® PLUS™ 0.76 (0.06) mm. For the ‘S’-type specimens thicknesses of 0.33 (0.03) mm (Duran Plus®), 0.45 (0.10) mm (Zendura®), 0.36 (0.04) mm (Essix ACE®), and 0.47 (0.05) mm (Essix® PLUS™) were measured. The variance for all thickness results of the as-received sheets was below 0.01. Detailed information on thickness reduction and percentage of thickness reduction can be found in the Supplemental Table 1.

### Forces of as-received sheets

**Fig. 4:**
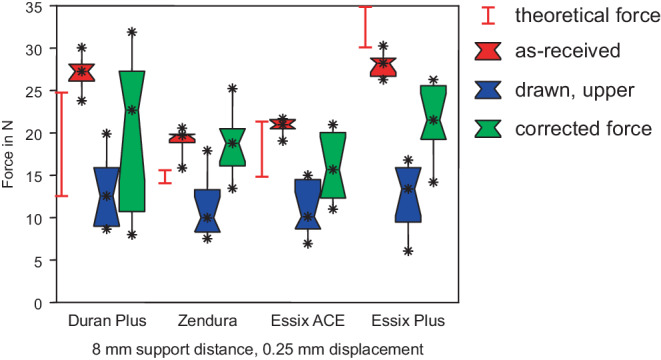
Forces in 3‑point bending test with a support distance of 8 mm. Duran Plus® and Essix® PLUS™ generated highest forces in as-received state. Significant force reduction for deep drawn foil sheets. The corrected forces do not reach the force of as-received specimens. *Red box* As-received, *blue box* drawn, *green box* corrected force. The range of the theoretical forces of as-received sheets is marked with the *red lines* Kräfte im 3‑Punkt-Biegeversuch mit einem Stützpunktabstand von 8 mm. Duran Plus® und Essix® PLUS™ erzeugten im unbehandelten Zustand die höchsten Kräfte. Deutliche Kraftreduzierung bei tiefgezogenen Folien. Die korrigierten Kräfte erreichen nicht die Kraft der unbehandelten Probe. *Rote Boxen* nicht tiefgezogen, *blaue Boxen* tiefgezogen, *grüne Boxen* rückgerechnete, korrigierte Kraft. Der Bereich der theoretischen Kräfte der unbehandelten Folien ist mit *roten Linien* markiert

**Fig. 5:**
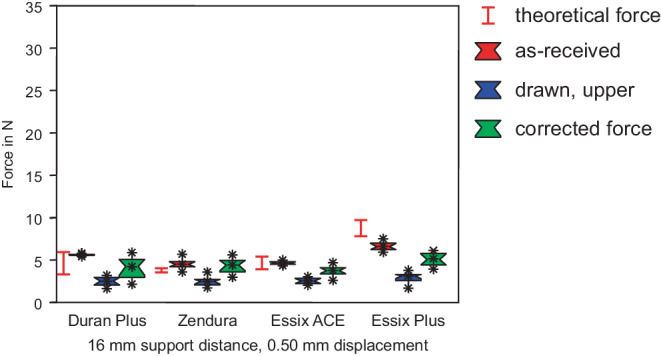
Forces in 3‑point bending, support distance of 16 mm for as-received specimens and specimens from the upper side. After thermoforming differences between the products did not prove to be significant. *Red box* As-received, *blue box* drawn, *green box* corrected force. The range of the theoretical forces of as-received sheets is marked with the *red lines* Kräfte im 3‑Punkt-Biegeversuch, Stützpunktabstand 16 mm für unbehandelte Proben und Proben aus der Oberseite. Nach dem Tiefziehen erwiesen sich die Unterschiede zwischen den verschiedenen Produkten als nicht signifikant. *Rote Boxen* nicht tiefgezogen, *blaue Boxen* tiefgezogen, *grüne Boxen* rückgerechnete, korrigierte Kraft. Der Bereich der theoretischen Kräfte der unbehandelten Folien ist mit *roten Linien* markiert

**Fig. 6:**
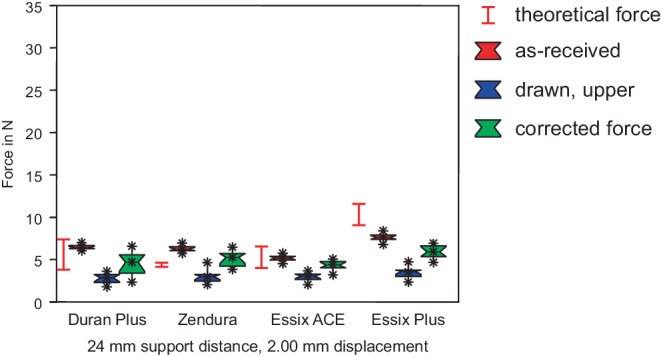
Forces in 3‑point bending, support distance of 24 mm, as-received specimens and specimens from the upper side. Highest forces are measured for Essix® PLUS™, significant decrease of forces for thermoformed sheets. Corrected forces did not reach the forces of the as-received specimens. *Red box* As-received, *blue box* drawn, *green box* corrected force. The range of the theoretical forces of as-received sheets is marked with the *red lines* Kräfte im 3‑Punkt-Biegeversuch, Stützpunktabstand 24 mm, unbehandelte Proben und Proben aus der Oberseite. Höchste Kräfte wurden für Essix® PLUS™ gemessen, signifikante Abnahme der Kräfte für tiefgezogene Folien. Die korrigierten Kräfte erreichten nicht die Kräfte der unbehandelten Proben. *Rote Boxen* nicht tiefgezogen, *blaue Boxen* tiefgezogen, *grüne Boxen* rückgerechnete, korrigierte Kraft. Der Bereich der theoretischen Kräfte der unbehandelten Folien ist mit *roten Linien* markiert

**Fig. 7:**
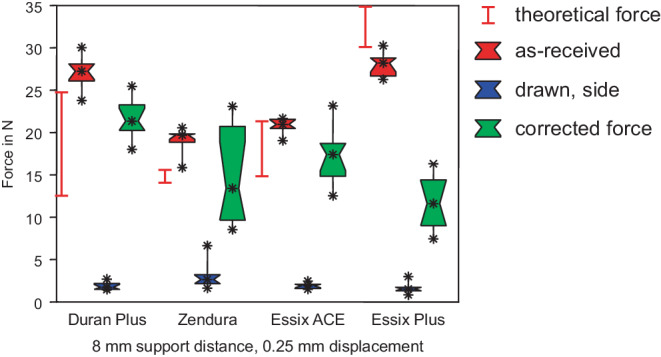
Forces in 3‑point bending, support distance of 8 mm, as-received specimens and specimens from the side walls. After deep drawing, differences between forces were not significant. *Red box* As-received, *blue box* drawn, *green box* corrected force. The range of the theoretical forces of as-received sheets is marked with the *red lines* Kräfte im 3‑Punkt-Biegeversuch, Stützpunktabstand 8 mm, unbehandelte Proben und tiefgezogene Proben von den Seitenwänden. Nach dem Tiefziehen waren die Unterschiede zwischen den Kräften nicht signifikant. *Rote Boxen* nicht tiefgezogen, *blaue Boxen* tiefgezogen, *grüne Boxen* rückgerechnete, korrigierte Kraft. Der Bereich der theoretischen Kräfte der unbehandelten Folien ist mit *roten Linien* markiert

**Fig. 8:**
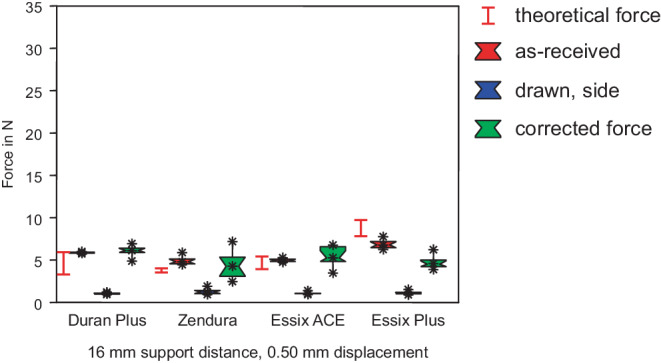
Forces in 3‑point bending, support distance of 16 mm, as-received specimens and specimens from the side. Significant decrease of forces for deep drawn sheets. *Red box* As-received, *blue box* drawn, *green box* corrected force. The range of the theoretical forces of as-received sheets is marked with the *red lines* Kräfte im 3‑Punkt-Biegeversuch, Stützpunktabstand 16 mm, unbehandelte Proben und tiefgezogene Proben von den Seitenwänden. Deutliche Reduzierung der Kräfte bei tiefgezogenen Folien. *Rote Boxen* nicht tiefgezogen, *blaue Boxen* tiefgezogen, *grüne Boxen* rückgerechnete, korrigierte Kraft. Der Bereich der theoretischen Kräfte der unbehandelten Folien ist mit *roten Linien* markiert

**Fig. 9:**
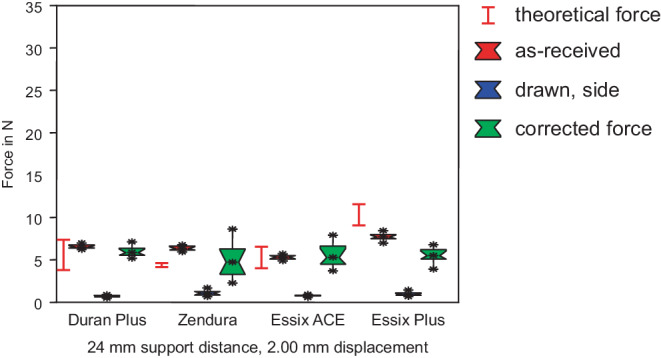
Forces in 3‑point bending, support distance of 24 mm, as-received specimens and specimens from the side. No significant differences for forces generated by thermoformed sheets. *Red box* As-received, *blue box* drawn, *green box* corrected force. The range of the theoretical forces of as-received sheets is marked with the *red lines* Kräfte im 3‑Punkt-Biegeversuch, Stützpunktabstand 24 mm, unbehandelte Proben und Proben von den Seitenwänden. Keine signifikanten Unterschiede bei den Kräften, die von tiefgezogenen Folien erzeugt werden. *Rote Boxen* nicht tiefgezogen, *blaue Boxen* tiefgezogen, *grüne Boxen* rückgerechnete, korrigierte Kraft. Der Bereich der theoretischen Kräfte der unbehandelten Folien ist mit *roten Linien* markiert

Figs. 4, 5 and 6 display the comparison of measured forces of as-received sheets with drawn specimens from the upper side, while Figs. 7, 8 and 9 display the comparison of as-received forces with forces of drawn specimens from the side walls. In all figures, the range of the theoretical forces is marked by red lines and red boxes represent the forces of as-received specimens (group I). Essix® PLUS™ generated the highest force of 28.2 N in 3‑point bending with 8 mm support distance and displacement of 0.25 mm (Figs. 4 and 7), corresponding to the largest thickness of 0.90 mm of this foil. Duran Plus® followed with a force of 27.3 N. The difference was not significant (Table 2). Essix ACE® (21.0 N) and Zendura® (19.7 N) generated significantly lower forces. Except for Essix® PLUS™ all theoretical forces are lower than actually measured forces of as-received foil sheets. Furthermore, they cover a typically extremely large range, indicating the high variance of Young’s modulus found in literature. This finding holds for all following box plots.

**Table 2:** Results of statistical test of all measured values at a support distance of 8 mm. Product name without additional remark means measured force in as-received state. “Upper” and “Side” mark forces of specimens cut from the drawn foil sheets at the upper side or the side walls Ergebnis der statistischen Prüfung der Messwerte bei einem Stützpunktabstand von 8 mm. Produktname ohne zusätzlichen Hinweis bedeutet gemessene Kraft im unbehandelten Zustand. „Upper“ und „Side“ kennzeichnen Kräfte von Proben, die aus den tiefgezogenen Folien der Oberseite oder den Seitenwänden geschnitten wurden

*Duran Upper*	s	–	–	–	–	–	–	–	–	–	–
*Side*	s	s	–	–	–	–	–	–	–	–	–
*Zendura*	s	0.00029	s	–	–	–	–	–	–	–	–
*Upper*	s	0.41699	s	s	–	–	–	–	–	–	–
*Side*	s	s	0.05238	s	s	–	–	–	–	–	–
*Essix ACE*	s	s	s	0.00915	s	s	–	–	–	–	–
*Upper*	s	0.43416	s	s	0.97403	s	s	–	–	–	–
*Side*	s	s	0.72760	s	s	0.06304	s	s	–	–	–
*Essix Plus*	0.19823	s	s	s	s	s	s	s	s	–	–
*Upper*	s	0.71183	s	0.00164	0.25552	s	0.00029	0.26694	s	s	–
*Side*	s	s	0.31963	s	s	0.02334	s	s	0.18854	s	s
*–*	*Duran*	*Upper*	*Side*	*Zendura*	*Upper*	*Side*	*Essix ACE*	*Upper*	*Side*	*Essix Plus*	*Upper*

*s* significant

At a support distance of 16 mm (Figs. 5 and 8), all foil sheets generated significantly reduced (Table 3) forces (Duran Plus® 5.6 N, Zendura® 4.5 N, Essix ACE® 4.7 N, Essix® PLUS™ 6.6 N) in spite of increased deflection. At a support distance of 24 mm (Figs. 6 and 9), forces ranged from 5.2 N (Essix ACE®) to 7.5 N (Essix® PLUS™). The difference between the forces generated by Duran® Plus™ (6.4 N) and Zendura® (6.2 N) was not significant (Table 4).

**Table 3:** Results of statistical test of all measured values at a support distance of 16 mm. Product name without additional remark means measured force in as-received state. “Upper” and “Side” mark forces of specimens cut from the drawn foil sheets at the upper side or the side walls Ergebnis der statistischen Prüfung der Messwerte bei einem Stützpunktabstand von 16 mm. Produktname ohne zusätzlichen Hinweis bedeutet gemessene Kraft im unbehandelten Zustand. „Upper“ und „Side“ kennzeichnen Kräfte von Proben, die aus den tiefgezogenen Folien der Oberseite oder den Seitenwänden geschnitten wurden

*Duran Upper*	s	–	–	–	–	–	–	–	–	–	–
*Side*	s	s	–	–	–	–	–	–	–	–	–
*Zendura*	s	s	s	–	–	–	–	–	–	–	–
*Upper*	s	0.76279	s	s	–	–	–	–	–	–	–
*Side*	s	s	0.01517	s	s	–	–	–	–	–	–
*Essix ACE*	s	s	s	0.67990	s	s	–	–	–	–	–
*Upper*	s	0.82244	s	s	0.88563	s	s	–	–	–	–
*Side*	s	s	0.13350	s	s	0.05202	s	s	–	–	–
*Essix Plus*	s	s	s	s	s	s	s	s	s	–	–
*Upper*	s	0.05477	s	s	0.13311	s	s	0.05932	s	s	–
*Side*	s	s	0.07567	s	s	0.14432	s	s	0.44767	s	s
*–*	*Duran*	*Upper*	*Side*	*Zendura*	*Upper*	*Side*	*Essix ACE*	*Upper*	*Side*	*Essix Plus*	*Upper*

*s* significant

**Table 4:** Results of statistical test of all measured values at a support distance of 24 mm. Product name without additional remark means measured force in as-received state. “Upper” and “Side” mark forces of specimens cut from the drawn foil sheets at the upper side or the side walls Ergebnis der statistischen Prüfung aller Messwerte bei einem Stützabstand von 24 mm. Produktname ohne zusätzlichen Hinweis bedeutet gemessene Kraft im unbehandelten Zustand. „Upper“ und „Side“ kennzeichnen Kräfte von Proben, die aus den tiefgezogenen Folien der Oberseite oder den Seitenwänden geschnitten wurden

*Duran Upper*	s	–	–	–	–	–	–	–	–	–	–
*Side*	s	s	–	–	–	–	–	–	–	–	–
*Zendura*	0.10716	s	s	–	–	–	–	–	–	–	–
*Upper*	s	0.35678	s	s	–	–	–	–	–	–	–
*Side*	s	s	0.00369	s	s	–	–	–	–	–	–
*Essix ACE*	s	s	s	s	s	s	–	–	–	–	–
*Upper*	s	0.49454	s	s	0.65781	s	s	–	–	–	–
*Side*	s	s	0.13130	s	s	0.01701	s	s	–	–	–
*Essix Plus*	s	s	s	s	s	s	s	s	s	–	–
*Upper*	s	0.01128	s	s	0.14082	s	s	0.02974	s	s	–
*Side*	s	s	0.00682	s	s	0.29416	s	s	0.05920	s	s
*–*	*Duran*	*Upper*	*Side*	*Zendura*	*Upper*	*Side*	*Essix ACE*	*Upper*	*Side*	*Essix Plus*	*Upper*

*s* significant

### Forces of deep drawn sheets

The force measurements of group II showed the influence of heat treatment and deep drawing. This process resulted in thickness reduction. Forces of specimens cut from the upper side are shown in Figs. 4, 5, and 6, and results for specimens cut from the side walls are shown in Figs. 7, 8, and 9. Forces of drawn sheets are shown in blue.

### Specimens from upper side

At 8 mm support distance (Fig. 4) Essix® PLUS™ generated the highest force of 13.4 N, followed by Duran Plus® (12.6 N), Essix ACE® (10.1 N), and Zendura® (10.0 N). All the measured differences between forces of the different drawn sheets were not statistically significant. However, forces were reduced significantly compared to the as-received forces (Table 2).

As for the as-received specimens, forces decreased significantly with the increase of the support distance to 16 mm (Fig. 5). Forces ranged from 2.5 N (Duran Plus®, Zendura®, Essix ACE®) to 3.2 N (Essix® PLUS™). Again, all differences between forces of drawn sheets were not significant; however, forces were reduced significantly compared to the as-received forces (Table 3). Similar behavior was seen for a support distance of 24 mm (Fig. 6; Table 4).

### Specimens from side walls

The thickness of specimens from side walls was further reduced compared to specimens from the upper side (Fig. 3). Thus, a drastic decrease of the forces according to bh^3^ (see Eq. 2) became obvious (Figs. 7, 8 and 9). Forces decreased below 2 N for a support distance of 8 mm, and below 1 N for 16 and 24 mm support distances. All the measured differences between forces generated by the thermoformed sheets were not statistically significant, while the differences between as-received and thermoformed sheets were significant (Tables 2, 3 and 4).

### Influence of support distances

In order to quantify and compare the influence of the tested support distances, the percentage changes of measured forces were calculated and listed in Table 5. Force reduction for specimens cut from the upper side is around 50%, for specimens from side walls around 90% for all three test configurations. Obviously, there is only a minor influence from the support distance of the 3‑point bending test. Differences between forces measured at the different support distances were not statistically significant, neither for the individual materials nor for the mean.

**Table 5:** Results of statistical test of recalculated Young’s moduli. Product name without additional remark means measured force in as-received state. “Upper” and “Side” mark forces of specimens cut from the drawn foil sheets at the upper side or the side walls Ergebnisse des statistischen Tests der neu berechneten Elastizitätsmodule. „Upper“ und „Side“ kennzeichnen Kräfte von Proben, die aus den tiefgezogenen Folien der Oberseite oder den Seitenwänden geschnitten wurden

*Duran Upper*	s	–	–	–	–	–	–	–	–	–	–
*Side*	s	0.02179	–	–	–	–	–	–	–	–	–
*Zendura*	s	0.90686	s	–	–	–	–	–	–	–	–
*Upper*	s	0.20066	s	0.00946	–	–	–	–	–	–	–
*Side*	s	0.04239	s	0.00378	0.16969	–	–	–	–	–	–
*Essix ACE*	s	0.57477	s	0.03629	s	0.00032	–	–	–	–	–
*Upper*	s	0.03474	s	s	0.09807	0.74874	s	–	–	–	–
*Side*	s	0.70869	0.00304	0.37805	0.01163	0.00239	0.81170	s	–	–	–
*Essix Plus*	s	0.04569	s	s	0.11543	0.51544	s	0.56211	s	–	–
*Upper*	s	s	s	s	s	0.01152	s	s	s	s	–
*Side*	s	s	s	s	s	s	s	s	s	s	s
*–*	*Duran*	*Upper*	*Side*	*Zendura*	*Upper*	*Side*	*Essix ACE*	*Upper*	*Side*	*Essix Plus*	*Upper*

*s* significant

### Young’s modulus and corrected forces

Force reduction due to thickness reduction was compensated using formula (1). Corrected forces are displayed in green (Figs. 4, 5, 6, 7, 8 and 9). Obviously, except for several selected examples, the corrected forces could not completely predict the force reduction due to thickness reduction.

**Fig. 10:**
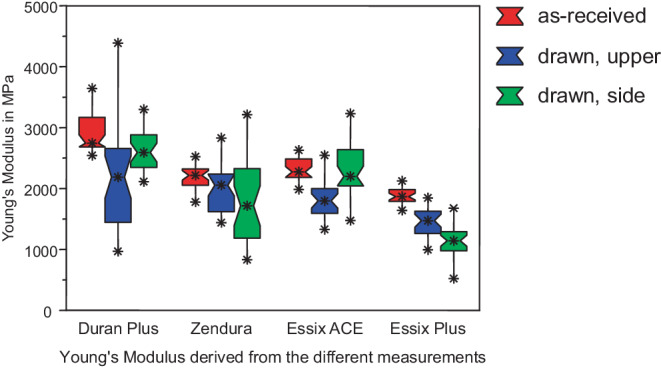
Young’s modulus of as-received and deep drawn foil sheets (*red*: as-received, *blue*: upper side, *green*: side walls). Obviously, Young’s modulus decreases due to thermoforming Elastizitätsmodul von unbehandelten und tiefgezogenen Alignerfolien. (*rot*: unbehandelt, *blau*: tiefgezogen, Oberseite, *grün*: tiefgezogen, Seitenwände). Offensichtlich nimmt das Elastizitätsmodul aufgrund der Wärmebehandlung ab

Fig. 10 shows the Young’s moduli (E) calculated from all measurements for as-received and thermoformed specimens. Essix® PLUS™ had the lowest Young’s modulus of 1869 MPa and showed a gradual and statistically significant decrease of Young’s modulus with thickness reduction to 1473 and 1144 MPa for specimens cut from the upper side and side walls, respectively. Young’s modulus of Duran Plus® (2746 MPa) and Essix ACE® (2274 MPa) first decrease (from as-received to upper side specimens: 2189 and 1798 MPa) and then increased again for specimens from the side walls (2592 and 2201 MPa). Reductions of E from as-received to deep drawn sheets for Duran® Plus™ were statistically significant, while the difference between as-received sheet and specimen from side wall of Essix ACE® was not significant. Zendura® showed a gradual (2218, 2057, 1718 MPa) but not significant decrease of E (Table 6).

**Table 6:** Results of statistical test of recalculated Young’s moduli. Product name without additional remark means measured force in as-received state. “Upper” and “Side” mark forces of specimens cut from the drawn foil sheets at the upper side or the side walls Ergebnis des statistischen Tests der rückgerechneten Elastizitätsmodule. Produktname ohne zusätzlichen Hinweis bedeutet gemessene Kraft im unbehandelten Zustand. „Upper“ und „Side“ kennzeichnen Kräfte von Proben, die aus den tiefgezogenen Folien der Oberseite oder den Seitenwänden geschnitten wurden

*Duran* *Upper*	s	–	–	–	–	–	–	–	–	–	–
*Side*	s	0.02179	–	–	–	–	–	–	–	–	–
*Zendura*	s	0.90686	s	–	–	–	–	–	–	–	–
*Upper*	s	0.20066	s	0.00946	–	–	–	–	–	–	–
*Side*	s	0.04239	s	0.00378	0.16969	–	–	–	–	–	–
*Essix ACE*	s	0.57477	s	0.03629	s	0.00032	–	–	–	–	–
*Upper*	s	0.03474	s	s	0.09807	0.74874	s	–	–	–	–
*Side*	s	0.70869	0.00304	0.37805	0.01163	0.00239	0.81170	s	–	–	–
*Essix Plus*	s	0.04569	s	s	0.11543	0.51544	s	0.56211	s	–	–
*Upper*	s	s	s	s	s	0.01152	s	s	s	s	–
*Side*	s	s	s	s	s	s	s	s	s	s	s
*–*	*Duran*	*Upper*	*Side*	*Zendura*	*Upper*	*Side*	*Essix ACE*	*Upper*	*Side*	*Essix Plus*	*Upper*

*s* significant

## Discussion

Aligner fabrication by thermoforming results in significant thickness reduction, as shown by the presented results and previous studies [3, 8, 14, 21]. Further aspects of material alterations affected by thermoforming and/or mechanical or thermal cycling during intraoral use are increased water solubility and absorption, or a decreased transparency [3, 8, 21]. A combined mechanical loading and water storage of aligner foil sheets results in force reduction of up to 50% after 24 h due to relaxation [8, 15]. However, the most relevant factors seem to be the thermoforming process, with the resulting thickness reduction and the change to the complex aligner geometry, resulting in significant force alterations. Thus, this aspect is of highest relevance with respect to clinical application as forces on the individual teeth may be affected drastically.

Thickness reduction upon thermoforming was extremely inhomogeneous and depended on the underlying geometry (compare Fig. 3): Reduction on the side walls was much higher than on the upper side of the master plate. This can be compared to the situation during thermoforming using clinical casts with the occlusal surfaces of molars and premolars and the buccal/lingual sides of the individual teeth. Material thickness will be much less on the vertical segments of an aligner. Elkholy et al. [8] also reported on the influence of the form used for thermoforming on thickness reduction and force delivery. Depending on the geometry they found thickness reduction between 8 and 17% for flat surfaces and a gable roof form, respectively. Clinically this leads to significantly reduced material stiffness on the buccal and lingual sides of aligners with possible reduced control with respect to certain tooth movements (e.g., rotation, tipping or torque).

As already pointed out by Elkholy et al. [8], a 3-point bending test does not take into account the complex geometry of a thermoformed aligner and the deformation behavior of aligners during intraoral application. However, standardized testing of an aligner with its complex 3D geometry in a biomechanical set-up on models of dental arches is highly complex and comparison of different materials and analysis of the effect of thermoforming on the different materials is complicated in such situations. We thus decided to perform tests with the simple and standardized geometry of the 3‑point bending set-up as it allows comparing different materials and material alterations directly without the effect of geometrical changes. Although forces measured in 3‑point bending tests do not have a direct clinical significance, this standardized and simple test allows to document material alterations of foil sheets due to thermoforming. Clinically relevant findings from this study are that the flexure modulus seems to change and that larger vertical steps at side walls result in extreme thickness reduction. Both factors can alter the ability of an aligner to transmit controlled forces and moments on a tooth.

One critical aspect of this study and some similar studies [14, 21] could be that experiments have been performed at room temperature. As reported by Ihsen et al. [10], the ambient temperature of experimental testing of foil sheets is of importance and tests should be done at 37 °C, as Young’s modulus of thermoplastic materials decrease with increasing temperature. Consequently, tests were performed at an ambient temperature of 37 °C in the study presented by Elkholy et al. [8]. Indeed this reduced Young’s modulus has an effect during testing of a full aligner in a biomechanical set-up; however, it may be assumed that material alterations due to thermoforming or force changes due to thickness reduction are similar, irrespective of the ambient temperature.

As shown in this study, thermoforming resulted in significant reduction of forces of all tested materials. This conforms to a series of earlier studies [3, 8, 10, 14]. Elkholy et al. [8] determined force reductions of up to 75% after drawing the foil sheets. This reduction was basically attributed to thickness changes and was comparable to the reduction we determined for the side walls. However, we showed that the force reduction was not only caused by thickness reduction, but that thermoforming changed the mechanical properties of the polymer material as well. Although the reduction of Young’s modulus was not uniform or consistent, we could see a decrease of Young’s modulus for all thermoformed compared to as-received materials. Most differences between deep drawn and as-received specimens were statistically significant. A similar behavior was reported by Ryu et al. [21] for flexure and elastic modulus, while Ihsen et al. [10] reported a statistically significant reduction of Young’s modulus after 24 h immersion in distilled water and/or ageing by thermocycling.

Young’s moduli derived from flexure and tensile tests by Ryu et al. [21] and Tamburrino et al. [26] differ slightly from our values. Selected specimens showed an increase in Young’s modulus after thermoforming with a slight tendency to a general decrease, while we determined a decrease for all tested specimens. Thus, it can be concluded that the mechanical properties of aligner foil sheets decrease due to thermoforming and ageing. This decrease may be attributed to partial changes from the amorphous to the crystalline structure of the thermoplastic material during thermoforming, where the crystalline phase might affect the elastic properties of the material [4, 21].

This in vitro study used an idealized 3‑point bending test, employing three different support distances simulating different spans of aligners in clinical situations, similar to studies of Kwon et al. [14] and Elkholy et al. [8]. It has to be clearly stated that this cannot represent the clinical situation of aligners on the whole dental arch. From mechanical point of view, aligners have an extremely complex geometry, following the morphology of the individual teeth with varying curvatures. Due to this geometry, aligners are much stiffer than thin, flat specimens are.

Furthermore, typical clinical deformations of aligners are clearly smaller than the deflections measured in our 3‑point bending test and are represented by the “staging” of aligner series of typically 0.2 mm. Consequently, the 3‑point bending test is a useful tool to characterize aligner foil sheets, but cannot be used to determine clinical force systems delivered by aligners. These clinical force systems have to be determined either in experimental biomechanical set-ups, simulating clinical situations or in combined clinical/biomechanical studies, as reported by Elkholy et al. [7, 19] or Simon et al. [24, 25].

## Conclusions

- Forces of foil sheets within the same brand did not differ markedly, both in the as-received and thermoformed state.
- The thickest material did not generate the highest force in all situations, especially after deep drawing, indicating that this material has a reduced Young’s modulus.
- Force reduction cannot be completely ascribed to thickness changes after thermoforming. Young’s modulus is slightly changed by heat treatment.
- The 3‑point bending test with varying support distances is an appropriate method to compare characteristics of aligner materials and the effect of thermoforming. The actual support distance is of minor importance.
- Clinically active forces, however, cannot be determined using this method.

## Supplementary Information

Supplemental Table 1 Measured thickness (mean and standard deviation) of the tested specimens from upper (‘U’) and side walls (‘S’) and the percentage changes of thickness reduction. Values listed are means and standard deviations from thickness measurements
