# Supplementary material for: Variation of the modulus of elasticity of aligner foil sheet materials due to thermoforming
Source: J Orofac Orthop. 2021 Aug 19;83(4):233–43. doi: 10.1007/s00056-021-00327-w (PMC9225978; doi:10.1007/s00056-021-00327-w)
Supplement: Supplementary file 1 — Supplemental Table 1 Measured thickness (mean and standard deviation) of the tested specimens from upper (‘U’) and side walls (‘S’) and the percentage changes of thickness reduction. Values listed are means and standard deviations from thickness measurements [file 56_2021_327_MOESM1_ESM.pdf]

Supplemental Table 1. Measured thickness (mean and standard deviation) of the tested specimens from upper (‘U’) and side walls (‘S’) and the percentage changes of thickness reduction. Values listed are means and standard deviations from thickness measurements.

Ergänzende Tabelle 1. Gemessene Dicke (Mittelwert und Standardabweichung) der Probekörper aus Oberseite (‘U’) und Seitenwänden (‘S‘) und prozentuale Änderungen der Dickenreduktion. Die aufgeführten Werte sind Mittelwerte und Standardabweichungen der Dickenmessungen

Product Name	Thickness as-received / mm	Thickness "U" / mm	Thickness "S" / mm	Thickness reduction "U" / %	Thickness reduction "S" / %
Duran Plus®	0.75	0.63 (0.05)	0.33 (0.03)	16.0	56.0
Zendura®	0.77	0.67 (0.05)	0.46 (0.03)	12.9	40.3
Essix ACE®	0.75	0.68 (0.06)	0.36 (0.05)	9.3	52.0
Essix® PLUS™	0.89	0.76 (0.07)	0.47 (0.03)	14.6	47.2
